# Association of glaucoma and lifestyle with incident cardiovascular disease: a longitudinal prospective study from UK Biobank

**DOI:** 10.1038/s41598-023-29613-w

**Published:** 2023-02-15

**Authors:** Jin A Choi, Su-Nam Lee, Sang-Hyuk Jung, Hong-Hee Won, Jae-Seung Yun

**Affiliations:** 1 Department of Ophthalmology, College of Medicine, St. Vincent’s Hospital, The Catholic University of Korea, Seoul, Republic of Korea; 2 Department of Internal Medicine, St. Vincent’s Hospital, College of Medicine, The Catholic University of Korea, 222 Banpo-daero, Seocho-gu, Seoul, 06591 Republic of Korea; 3 Samsung Advanced Institute for Health Sciences and Technology (SAIHST), Sungkyunkwan University, Samsung Medical Center, 81 Irwon-ro, Gangnam-gu, Seoul, 06351 Republic of Korea; 4 Samsung Genome Institute, Samsung Medical Center, Seoul, Republic of Korea

**Keywords:** Cardiology, Health care, Medical research, Risk factors, Ocular hypertension, Diseases, Eye diseases, Vision disorders

## Abstract

The shared pathophysiological features of the cerebrovascular disease (CVD) and glaucoma suggest an association between the two diseases. Using the prospective UK Biobank cohort, we examined the associations between glaucoma and incident CVD and assessed the extent to which a healthy lifestyle reduced the CVD risk in subjects with glaucoma, using a scoring system consisting of four factors: current smoking, obesity, regular physical activity, and a healthy diet. During a mean follow-up time of 8.9 years, 22,649 (4.9%) incident CVD cases were documented. Multivariable Cox regression analyses revealed that subjects with glaucoma were significantly more likely to exhibit incident CVD (hazard ratio [HR]:1.19, 95% confidence interval [CI] 1.03–1.37; *p* = 0.016) than controls. In the further subgroup analyses, glaucoma increased incident CVD risk both in the young (40–55 years) and the old (56–70 years) and in both sexes, with higher risk in the young (HR: 1.33, CI 1.02–1.74) and female subjects (HR: 1.32, CI 1.14–1.52). When we analyze the associations between glaucoma and incident CVD by lifestyle factors, the highest absolute risks were observed in individuals with both glaucoma and an unhealthy lifestyle (HR: 2.66, CI 2.22–3.19). In conclusion, glaucoma was an independent risk factor for incident CVD. A healthy lifestyle was associated with a substantially lower risk for CVD incidence among adults with glaucoma.

## Introduction

Glaucoma is the second-leading cause of blindness and the most common cause of irreversible blindness worldwide^1^. The glaucoma prevalence increased by 10.7% from 1990 to 2020, and continues to be a major cause of vision impairment, particularly in the aged^2^. A growing body of literature indicates that glaucoma is associated with many systemic vascular comorbidities^3–12^. Thus, the visual impairment of glaucoma imposes significant social burdens worldwide as the average age and life expectancy increase. Glaucoma pathogenesis features vascular involvement that dysregulates ocular perfusion and mechanical stress associated with intraocular pressure (IOP)^11^. The underlying mechanism of vascular dysregulation comprises disturbed autoregulation, endothelial dysfunction, and insufficient ocular blood flow associated with atherosclerosis^3,6–8,10,11^. Great deal of evidence has shown that patients with glaucoma exhibit a systemic circulatory disturbance as well as local vascular disturbance around the optic nerve head^11,13^.

Cardiovascular disease (CVD) is the leading cause of death worldwide. The subclinical pathophysiological features of CVD are also those of glaucoma, suggesting an association between CVD and glaucoma. Glaucoma patients are at a higher risk for death from CVD than are individuals without glaucoma^14^. Patients with glaucoma exhibit significantly higher prevalence of CVD and other comorbidities than age- and sex-matched controls^15^. The incident CVD rate is determined both by genetic factors and lifestyle behaviors. Unhealthy lifestyle behaviors are closely related to increased blood pressure, blood glucose and lipid levels, and the rate of being overweight or obese^16^, whereas a healthy lifestyle markedly reduces the risk for incident cardiovascular events^17,18^. Such a lifestyle is associated with a 50% lower risk for ischemic heart disease compared to an unhealthy lifestyle^19^. It is thus important to identify risk factors for the development of CVD in consideration of lifestyle behavioral factors. Here, we primarily investigated whether glaucoma is associated with an increased CVD incidence in a large, prospective, UK Biobank cohort. We further investigated the association between glaucoma and CVD according to age, sex, and IOP status. We then determined the extent to which a healthy lifestyle is associated with a reduced risk of CVD among participants with glaucoma.

## Methods

We used the dataset of the UK Biobank cohort. This prospective cohort study recruited approximately 500,000 middle-aged UK residents aged 40–69 years from 2006 to 2010; their health-related outcomes were followed up^20^. The protocol is publicly available; the project design and measurement procedures of UK Biobank have been described^21^. A baseline survey was performed at enrollment; all participants gave extensive phenotypic information on demographics, lifestyle behaviors, and medical histories using either a self-administered electronic questionnaire or interviews with trained nurses. Physical measurements were performed, and blood and urine samples were collected. All participants gave written informed consent to linkage of the UK Biobank dataset to their health-related records including primary care records, hospital inpatient records, and cancer and death registry data^22^. We excluded 35,799 participants diagnosed with coronary artery disease (CAD), myocardial infarction (MI), or ischemic stroke prior to the index date. This study was ethically approved by the Northwest Multi-center Research Ethics Committee (June 17, 2011 [reference 11/NW/0382]; this was extended on May 13, 2016 [reference 16/NW/0274]). The study adhered to all relevant tenets of the Declaration of Helsinki. Use of the UK Biobank resource was approved (application no. 90981).

The primary outcome was new-onset CVD (CAD, MI, and ischemic stroke). CAD was defined based on the first recorded occurrence of disease and hospitalization records. MI and ischemic stroke were algorithmically defined by the UK Biobank^23^. To exclude multiple events, only the first CVD outcome was considered. Glaucoma was considered present if this was patient-reported on the questionnaire or during interview, or if the diagnostic code for glaucoma (H40) but not secondary glaucoma (H40.3, H40.4, H40.5, H40.6, and H42) was present on the first examination of the database^24^. Detailed definitions of glaucoma and CVD are provided in Supplemental Table S1. The IOPs of 103,143 participants were measured in their assessment centers. IOPs were measured once for each eye using an Ocular Response Analyzer (Reichert Corp., Philadelphia, PA). We derived the corneal compensated IOP, which is a linear combination of the inward and outward applanation tensions^25^. The IOP values of the right and left eyes were averaged. If data were available for only one eye, we used that IOP.

The initial information collected included age, sex, ethnicity, familial medical history, medications, and lifestyles. Blood pressure was measured using the HEM-70151 T digital blood pressure monitor (Omron, Hoofddorp, The Netherlands). Height was measured using a Seca 202 height measure (Seca, Birmingham, UK), and weight was measured with the BV-418 MA body composition analyzer (Tanita, Arlington Heights, IL). Definition of baseline dyslipidemia and hypertension is described in Supplemental Table S1. Four major lifestyle behaviors affecting cardiovascular health were assessed based on the American Heart Association guidelines: obesity, current smoking, regular physical activity, and eating habits^19,26^. Obesity was defined as a body mass index > 30 kg/m^2^ according to the World Health Organization international classification. All participants were classified as either ‘current smokers’ or ‘non-smokers’ (not current and never-smokers). Self-reported physical activity data were recorded at baseline. Regular physical activity was defined as moderate activity on more than 5 days per week or vigorous activity on more than 3 days per week. Eating habits were defined based on the recommendations for cardiovascular health; the recommendations consider fruits, vegetables, whole grains, fish, dairy products, refined grains, processed meats, unprocessed meats, and sugar-sweetened beverages. A diet pattern was considered healthy if participants fulfilled at least half of the recommendations as assessed by a food frequency questionnaire^27^. Such behaviors were categorized into three groups: unhealthy (0 or 1 healthy factor), intermediate (2 healthy factors), and healthy (≥ 3 healthy factors). The details are shown in Supplemental Table S2.

During baseline assessment, blood samples were obtained and processed in a standard manner^21^. The sampling and processing of blood and urine samples have previously been described^28^. The HbA1c level was determined via high-performance liquid chromatography on Bio-Rad Variant II Turbo analyzers (Bio-Rad Laboratories, Hercules, CA) and lipid marker was determined using Beckman Coulter AU5800 auto-analyzers (Beckman Coulter, Inc., Brea, CA) running the following assays: the hexokinase assay for HbA1c and the enzymatic selective protection assay for low-density lipoprotein (LDL) cholesterol. The frequency of missing values is listed in Supplemental Fig. S1. More details are available at https://www.ukbiobank.ac.uk.

The baseline clinical characteristics of study participants were compared using the independent t-test for continuous variables and the chi-square test for categorical variables. The incidences of CVD events are presented as the rates per 1,000 person-years. To evaluate the association between glaucoma and CVD, we used multivariable Cox regression analysis. Model 1 was adjusted for covariates including age, sex, and race; Model 2 was further adjusted for a familial history of CVD, body mass index, smoking, physical activity level, and eating habits; Model 3 was adjusted for confounders of Model 2 plus blood pressure, estimated glomerular filtration rate, HbA1c level, and LDL cholesterol level; and the final model, Model 4 was additionally adjusted for the use of aspirin, any anti-hypertensive agent, any lipid lowering agents, and any diabetes medication. The Cox’s models were stratified by age (≤ 55 years and > 55 years) and sex. To assess whether lifestyle behaviors modified the association between glaucoma and CVD, we assessed the multiplicative interactions between glaucoma and lifestyle behaviors in terms of the subsequent risk for CVD. We also performed analyses for the CVD incidence according to the IOP deciles. Follow-up commenced on the date of enrollment and ceased on the date of loss to follow-up, scheduled follow-up conclusion (January 31, 2018 for England and Wales; November 30, 2016 for Scotland), or the date of death, whichever occurred first. Participants for whom data were missing were excluded. Schoenfeld residuals and log minus log plots were used to evaluate the proportional hazard assumptions. All statistical tests were two-sided, and *P* < 0.05 was considered statistically significant. All statistical analyses were conducted using R version 3.9.0.

## Results

**Figure 1:**
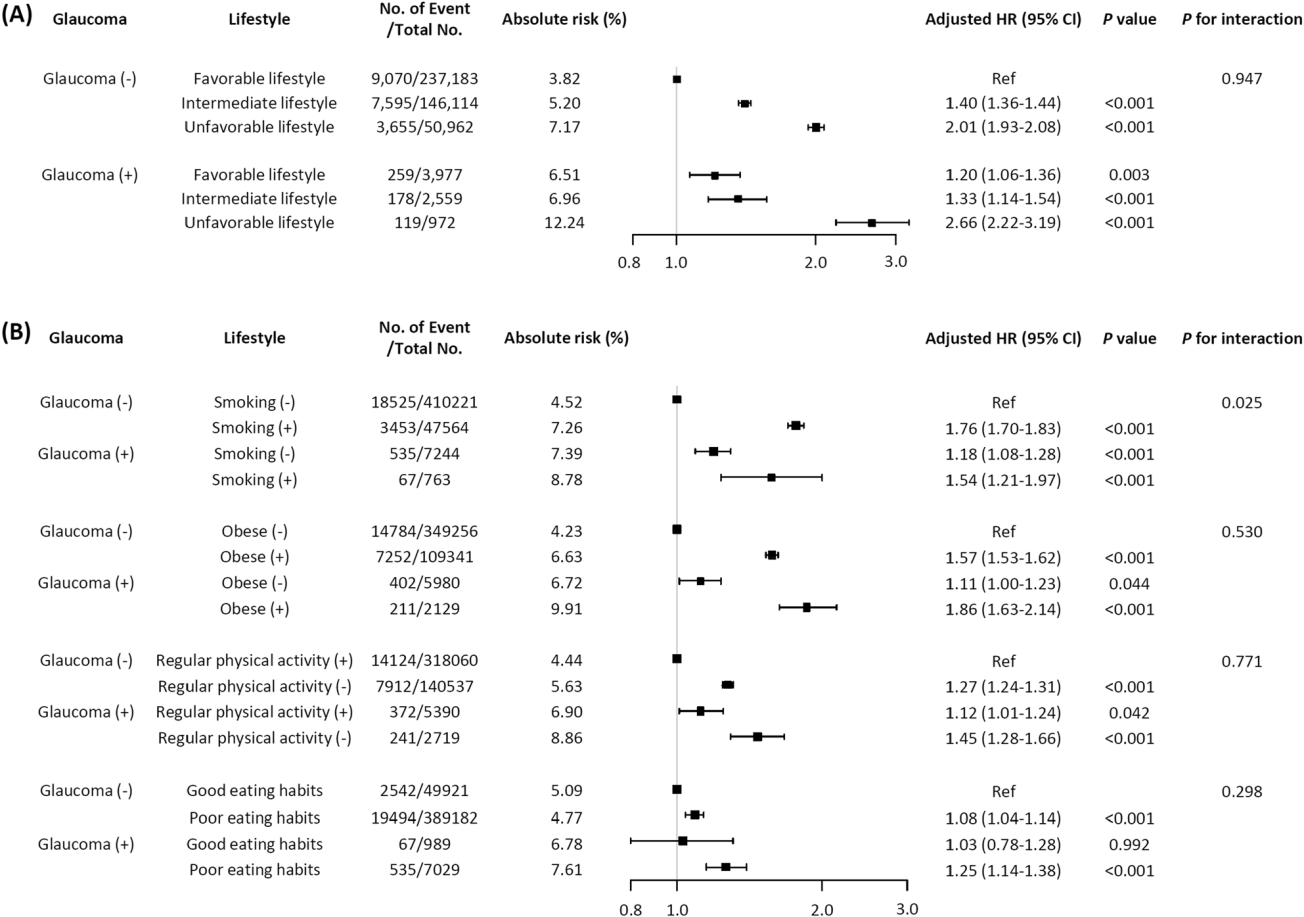
Forest plot for cardiovascular disease according to the presence of glaucoma and (**A**) lifestyle behavior, (**B**) smoking, obesity, physical activity, and eating habits. Results are shown after adjusting for age, sex, and race.

The sample sizes for eligible UK Biobank participants with complete data for our various analyses are presented in Supplemental Fig. S1. A total of 22,649 incident CVD cases (4.9%) were documented during a median follow-up of 8.9 years. The incidence of CVD was 5.61 per 1000 person-years (95% CI 5.53–5.68). Descriptive baseline characteristics of the 466,706 participants who met the inclusion criteria are listed in Table 1. In general, patients with glaucoma exhibited higher body mass index, blood pressure, HbA1c level, and incidences of hypertension, dyslipidemia, cancer, and unhealthy lifestyle behaviors than those without glaucoma (Table 1). Table 2 lists the HRs for incident CVD based on the univariable and multivariable Cox’s regression models. The unadjusted HR for CVD in participants with glaucoma was 1.60 compared with controls (95% CI 1.48–1.74, *P* < 0.001). After adjusting for family history, lifestyle factors, biometric measures, and the use of medications, participants with glaucoma were significantly more likely to develop incident CVD (Model 4, adjusted HR 1.19, 95% CI 1.03–1.37,* P* = 0.016) as revealed by multivariable regression analyses. In the further subgroup analyses, glaucoma increased incident CVD risk both in the young (40–55 years) and the old (56–70 years) and in both sexes, with higher risk in the young (HR: 1.33, CI 1.02–1.74) and female subjects (HR: 1.32, CI 1.14–1.52) (Supplemental Table S3). Figure 1 shows the associations between glaucoma and incident CVD by lifestyle factors. In both groups with and without glaucoma, an unhealthy lifestyle strongly predicted CVD; increased risk for CVD was seen in non-glaucomatous individuals with unhealthy lifestyle (absolute risk 7.17%, adjusted HR 2.01, 95% CI 1.93–2.08, *P* < 0.001), and the highest absolute risks were observed in individuals with both glaucoma and an unhealthy lifestyle (absolute risk 12.24%, adjusted HR 2.66, 95% CI 2.22–3.19, *P* < 0.001).

**Table 1:** Baseline characteristics of the study population.

	Total (N = 466,706)	Glaucoma (−) (N = 458,597)	Glaucoma (+) (N = 8109)	*P* value
Age	56.7 ± 8.1	56.6 ± 8.1	61.4 ± 6.6	< 0.001
Sex				
Female	261,030 (55.9)	257,048 (56.1)	3982 (49.6)	< 0.001
Male	205,584 (44.1)	201,548 (43.9)	4036 (50.4)	
Race				
White	439,174 (94.6)	431,730 (94.7)	7444 (92.3)	< 0.001
Asian	10,354 (2.2)	10,136 (2.2)	218 (2.7)	
Black	7580 (1.6)	7321 (1.6)	259 (3.2)	
Mixed	2805 (0.6)	2760 (0.6)	45 (0.6)	
Others	4253 (0.9)	4156 (0.9)	97 (1.2)	
Familial history of heart disease	185,923 (39.8)	182,576 (39.8)	3347 (41.3)	0.008
Familial history of stroke	117,448 (25.2)	115,128 (25.1)	2320 (28.6)	< 0.001
Body mass index (kg/m^2^)	27.3 ± 4.8	27.3 ± 4.7	27.6 ± 4.8	< 0.001
Current smoking	48,340 (10.4)	47,564 (10.4)	776 (9.6)	0.019
Moderate to vigorous physical activity	323,450 (69.3)	318,060 (69.4)	5390 (66.5)	< 0.001
Poor eating habits	50,923 (10.9)	49,921 (10.9)	1002 (12.4)	< 0.001
Lifestyle habits				
Healthy	241,195 (54.6)	237,183 (54.6)	4012 (52.8)	0.001
Intermediate	148,708 (33.7)	146,114 (33.6)	2594 (34.2)	
Unhealthy	51,949 (11.8)	50,962 (11.7)	987 (13.0)	
Systolic blood pressure (mmHg)	139.6 ± 19.7	139.6 ± 19.7	144.7 ± 20.0	< 0.001
Diastolic blood pressure (mmHg)	82.4 ± 10.7	82.4 ± 10.7	83.3 ± 10.7	< 0.001
HbA1c (%)	5.4 ± 0.6	5.4 ± 0.6	5.6 ± 0.7	< 0.001
LDL cholesterol (mg/dL)	139.3 ± 32.8	139.3 ± 32.8	138.9 ± 34.5	0.275
Estimated GFR (ml/min/1.73m^2^)	79.5 ± 14.3	79.5 ± 14.3	76.9 ± 14.1	< 0.001
Hypertension	123,299 (26.4)	120,233 (26.2)	3066 (37.8)	< 0.001
Dyslipidemia	66,165 (14.2)	64,312 (14.0)	1853 (22.9)	< 0.001
Cancer	53,449 (11.5)	52,307 (11.4)	1142 (14.1)	< 0.001
Aspirin	44,573 (9.6)	43,329 (9.4)	1244 (15.3)	< 0.001
Anti-hypertensive medications	40,461 (8.7)	39,269 (8.6)	1192 (14.7)	< 0.001
Lipid lowering medications	33,750 (7.2)	32,727 (7.1)	1023 (12.6)	< 0.001
Anti-hyperglycemic medications	14,238 (3.1)	13,801 (3.0)	437 (5.4)	< 0.001

Data are n (%) or mean (SD).

*CVD* cardiovascular disease, *GFR* glomerular filtration rate.

**Table 2:** Hazard ratios and 95% confidential intervals for cardiovascular disease using Cox proportional regression model.

	No. of CVD events/total no	Incidence rate per 1000 person-year (95% CI)	Absolute risk (%)	Crude		Model 1		Model 2		Model 3		Model 4	
				HR (95% CI)	*P* value	HR (95% CI)	*P* value	HR (95% CI)	*P* value	HR (95% CI)	*P* value	HR (95% CI)	*P* value
Baseline glaucoma (−)	22,036/458,597	5.55 (5.48–5.63)	1.67	Ref		Ref		Ref		Ref		Ref	
Baseline glaucoma (+)	602/8,108	8.86 (8.17–9.60)	2.66	1.60 (1.48–1.74)	< 0.001	1.13 (1.04–1.23)	0.003	1.13 (1.03–1.23)	0.007	1.20 (1.05–1.38)	0.009	1.19 (1.03–1.37)	0.016

Model 1: Age + Sex + Race.

Model 2: Model 1 + Familial history of heart disease + Familial history of stroke + BMI + Current smoking + Physical activity + Eating habits.

Model 3: Model 2 + Systolic BP + Diastolic BP + LDL cholesterol + HbA1c + Estimated glomerular filtration rate.

Model 4: Model 3 + Aspirin + Anti-hypertensive agents + Anti-hyperglycemic agents + Lipid lowering medications.

*BP* blood pressure, *CI* confidence interval, *HR* hazard ratio.

When the association between glaucoma and CVD was analyzed by each lifestyle component, similar trends were observed: risk of incident CVD events was further increased in individuals with glaucoma when they were obese, did not undertake vigorous physical exercise, and had poor eating habits, while no such trend was observed in association with smoking (Fig. 1).

The incident CVD risks were significantly reduced in glaucoma patients who were not current smoker (HR 0.77, 95% CI 0.60–0.99, *P* = 0.042), not obese (HR 0.62, 95% CI 0.52–0.73, *P* < 0.001), and engaged in vigorous physical activity (HR 0.77, 95% CI 0.65–0.91, *P* = 0.002) compared with those with an unhealthy lifestyle (Supplemental Table S4). Particularly, among patients with glaucoma, individuals who were not obese evidenced a CVD incidence rate 40% lower than that of those who were obese (adjusted HR 0.62, 95% CI 0.52–0.73,* P* < 0.001).

Among the 103,143 participants for whom IOPs were available, the risk for incident CVD increased in an approximately linear manner across IOP deciles (*P* for trends < 0.001) (Supplemental Table S5). The trend was significant in individuals without glaucoma (*P* for trends < 0.001), but not in those with glaucoma (*P* for trends = 0.752) (Supplemental Tables S6 and S7).

## Discussion

In this large community-based population of more than 466,706 individuals, glaucoma was an independent risk factor for increased risk of new-onset CVD, particularly among females and younger participants. When patients with glaucoma adhered to a healthy behavioral lifestyle, the risk was notably reduced.

Only one longitudinal study has explored the risk for increased ischemic heart disease in patients with open-angle glaucoma using the Taiwan National Health Insurance Research Database^29^. Studies from Korea and Taiwan found increased stroke incidences in glaucoma patients at 10 and 5 years of follow-up, respectively^30,31^. However, to the best of our knowledge, no previous reports have provided evidence for the offsetting effect of lifestyle behavioral factors on the association between glaucoma and CVD. We used the UK Biobank data, one of the largest available resources allowing powerful evaluation of the relationship between glaucoma and new-onset CVD with adjustment for multiple confounding factors including medications and biometric measures.

In line with previous studies^29–31^, we found that glaucoma is highly associated with cardiometabolic components, including diabetes, hypertension, dyslipidemia, chronic renal failure, ischemic events, and stroke. A significant body of epidemiologic studies indicates the IOP to have significant positive correlations with cardiometabolic risk factors including body mass index, blood pressure, fasting blood glucose level, and lipid profile^32,33^. Indeed, we found that incident CVD risk tended to increase with IOP decile in all participants and in those without glaucoma, but the trend was not significant in individuals with glaucoma, which could be related to the use of anti-glaucoma medication in such patients. IOP is regulated by the balance between inflow and outflow of the aqueous humor in the anterior chamber of the eye. From a structural perspective, the anterior chamber could be considered a specialized circulatory vessel lined with endothelial cells of the corneal and trabecular endothelium^34^. In this regard, it is presumed that the close relationship between cardiometabolic components and IOP could in turn lead to significant association of the IOP with CVD development.

Previous studies have shown association of glaucoma with various cardiometabolic factors^35–40^. Glaucoma pathogenesis involves both vascular and mechanical mechanisms. In particular, disc hemorrhage, which plays a role in glaucoma development and progression, is strongly associated with vascular instability^11^. Vascular insufficiency around the optic nerve head caused by endothelial dysfunction, autonomic instability, and ischemic changes triggered by atherosclerosis have been suggested to aggravate injury to retinal ganglion cell axons subjected to mechanical compression at the level of the lamina cribrosa^8–10,13^. Notably, heart rate variability, which reflects autonomic system function^41^, is reportedly low in patients with glaucoma^3–5^. In the general population, individuals with low heart rate variability have adverse cardiovascular risk profiles and are at elevated risk for incident ischemic heart disease and death^42,43^. Thus, the subclinical risk factors shared by the two conditions may play important roles in the development of new-onset CVD in glaucoma patients.

A great deal of evidence has shown that healthy lifestyle behaviors (not smoking, avoiding obesity, regular physical activity, and a healthy diet) markedly reduce the rate of incident cardiovascular events^17,18^. Notably, we found that glaucoma patients with healthy lifestyle behaviors were at reduced risk for incident CVD compared to those with unhealthy behaviors. When analyzed in terms of individual lifestyle components, we found that not being obese, engaging in vigorous exercise, and eating a healthy diet reduced HR for incident CVD in glaucoma patients. Of these, obesity had the strongest effect, while smoking habits had a rather attenuated effect on CVD risk. It is well known that smoking is an important risk factor for virtually all CVD subtypes, at least doubling risk in the case of a current smoker^44^. A recent systematic review also reported that heavy smoking as well as current smoking significantly increases the risk of primary open angle glaucoma (POAG,) although there is limited evidence for a causal association between tobacco smoking and POAG^45^. The specific mechanism underlying the association between glaucoma, smoking, and cardiovascular disease is yet unclear. Overall, this finding suggests that the association of glaucoma with CVD may considerably overlap with an underlying, as-yet-unidentified pathway linked to smoking. Further studies remain needed to elucidate these relationships. Epidemiological studies on lifestyle factors have reported no clear association of any environmental factor with glaucoma^46^. However, considering the high mortality burden from CVD, patients with glaucoma should pursue ideal lifestyles to minimize the risk of future CVD events.

Notably, glaucoma was associated with higher incident CVD risk in women and younger participants, although CVD events were significantly more common in males and older individuals (*P* < 0.001, Table 1). The greater influence of glaucoma in a traditionally low-risk CVD group may indicate an independent effect of glaucoma on CVD development. Women with glaucoma are at greater risk for CVD than the general population, highlighting the importance of CVD prevention in these population^47^. Further studies are required to elucidate these findings.

To the best of our knowledge, this is the first study to investigate the association between glaucoma and incident CVD with consideration of modifiable health behaviors. The major strengths of the present study are the large sample size and the prospective study design. However, our study had certain limitations. We did not divide glaucoma into subtypes. Although we included both self-reported glaucoma and glaucoma based on the ICD diagnostic codes as done in previous reports^24^, the definition of self-reported glaucoma might not be very specific. Second, as this was an observational study, any causal link among glaucoma, lifestyle behaviors, and CVD risk cannot be inferred. Third, the UK Biobank is a cohort of volunteers; overall participants are probably healthier than the general population. Fourth, the proportion of participants with current smoking in the glaucoma group was relatively less than other subgroups, which could have affected the association between smoking and risk of incident CVD. Finally, our quality control process excluded participants, perhaps creating selection bias. Finally, our quality control process excluded participants, perhaps creating selection bias.

In conclusion, we present a large epidemiological study of the association between glaucoma and CVD. Glaucoma was an independent risk factor for incident CVD. Stronger associations were evident in women and those younger than 55 years. Glaucoma patients with a healthy lifestyle were at a markedly reduced risk for new-onset CVD. Our findings further support benefits of adherence to a healthy lifestyle in reducing subsequent burden of cardiovascular complications in patients with glaucoma.

## Supplementary Information

Supplementary Information.

## Data Availability

The UK Biobank dataset was obtained from the UK Biobank (Application Number: 90981), and a full list of the variables are available online (http://biobank.ndph.ox.ac.uk/showcase/). Data cannot be shared publicly due to violation of the regulation of UK Biobank and patient privacy and the absence of informed consent for data sharing. Individual level data for UK Biobank participants are available to eligible researchers through the UK Biobank (www.biobank.ac.uk). For details please contact access@ukbiobank.ac.uk.
