# Supplementary material for: Association of glaucoma and lifestyle with incident cardiovascular disease: a longitudinal prospective study from UK Biobank
Source: Sci Rep. 2023 Feb 15;13:2712. doi: 10.1038/s41598-023-29613-w (PMC9931750; doi:10.1038/s41598-023-29613-w)
Supplement: Supplementary file 1 — Supplementary Information. [file 41598_2023_29613_MOESM1_ESM.docx]

**Supplemental Materials**

**Supplemental Table 1. Detailed definitions of glaucoma, cardiovascular disease, cardiovascular outcomes and comorbidities**

| **Disease** | **Path** | **Field ID** | **Code** |
| --- | --- | --- | --- |
| Baseline glaucoma | Touchscreen | Eye problems/disorders (6148) | Glaucoma |
|  | Verbal interview | Non-cancer illness, self-report (20002) | Glaucoma (1277) |
|  | First occurrence before enrollment | First report of glaucoma (131186, 131187) | H40.x |
|  | First occurrence before enrollment for exclusion of secondary glaucoma | First report of glaucoma in diseases classified elsewhere (131186, 131187) | H42.x |
| Baseline coronary artery disease | Verbal interview | Non-cancer illness, self-report (20002) | 1074 (angina) |
|  |  |  | 1075 (heart attack/myocardial infarction) |
|  | Touchscreen | Vascular/heart problems diagnosed by doctor (6150) | Heart attack, angina |
|  | First occurrence before enrollment | First reported of angina pectoris (131296, 131297) | I20.x |
|  |  | First reported of acute myocardial infarction (131298, 131299) | I21.x |
|  |  | First reported of subsequent myocardial infarction (131300, 131301) | I22.x |
|  |  | First reported of certain current complications following acute myocardial infarction (131302, 131303) | I23.x |
|  |  | First reported of other acute ischemic heart diseases (131304, 131305) | I24.x |
|  |  | First reported of chronic ischemic heart disease (131306, 131307) | I25.x |
| Baseline ischemic stroke | Verbal interview | Non-cancer illness, self-report (20002) | Stroke (1081) |
|  |  |  | Ischemic stroke (1583) |
|  | Touchscreen | Vascular/heart problems diagnosed by doctor (6150) | Stroke |
|  | First occurrence before enrollment | First reported of cerebral infarction (131366, 131367) | I63.x |
|  |  | First reported of stroke, not specified as hemorrhage or infarction (131368, 131369) | I64.x |
| Cardiovascular outcome (coronary artery disease) | First occurrence after enrollment | First reported of angina pectoris (131296, 131297) | I20.x |
|  |  | First reported of acute myocardial infarction (131298, 131299) | I21.x |
|  |  | First reported of subsequent myocardial infarction (131300, 131301) | I22.x |
|  |  | First reported of certain current complications following acute myocardial infarction (131302, 131303) | I23.x |
|  |  | First reported of other acute ischemic heart diseases (131304, 131305) | I24.x |
|  |  | First reported of chronic ischemic heart disease (131306, 131307) | I25.x |
| Cardiovascular outcome (myocardial infarction) | UK Biobank algorithmically defined outcomes | Myocardial infarction outcomes (42000, 42001) |  |
| Cardiovascular outcome (ischemic stroke) | UK Biobank algorithmically defined outcomes | Ischemic stroke outcomes (42008, 42009) |  |
| Baseline dyslipidemia | Verbal interview | Non-cancer illness, self-report (20002) | High cholesterol (1473) |
|  | First occurrence before enrollment | First reported of disorders of lipoprotein metabolism and other lipidemia (130815, 130816) | E78.x |
|  | Medication | Medication for cholesterol, blood pressure or diabetes (6177) | Cholesterol lowering medication |
| Baseline hypertension | Verbal interview | Non-cancer illness, self-report (20002) | 1065, 1072 |
|  | Touchscreen | Vascular/heart problems diagnosed by doctor (6150) | High blood pressure |
|  | First occurrence before enrollment | First reported of essential hypertension (131286, 131287) | I10.x |
|  |  | First reported of hypertensive heart disease (131288, 131289) | I11.x |
|  |  | First reported of hypertensive renal disease (131290, 131291) | I12.x |
|  |  | First reported of hypertensive heart and renal disease (131292, 131293) | I13.x |
|  |  | First reported of secondary hypertension (131294, 131295) | I15.x |
|  | Medication | Medication for cholesterol, blood pressure or diabetes (6177) | Blood pressure medication |

**Supplemental Table 2. Detailed definitions of lifestyle factors and lifestyle behavior for cardiovascular risk**

| **Lifestyle factors** | **Component** | **Healthy lifestyle or metabolic status** | **Field ID of UK Biobank** |
| --- | --- | --- | --- |
| Current smoking | Current smoking at baseline | Absence | 20116 |
| Obesity | BMI at baseline | <30 kg/m^2^ | 21001 |
| Physical activity | Number of days per week of physical activity 10+ minutes | Participating in moderate activity ≥5 days a week or vigorous activity ≥3 days a week | 884 (Moderate physical activity 10+ minutes) 904 (Vigorous physical activity 10+ minutes) |
| Eating habits | At least half of all following diet components was considered as a healthy lifestyle, less than half was considered as an unhealthy lifestyle | | |
|  | Fruit | ≥3 serving/day | 1309 (Fresh fruit) 1319 (Dried fruit) |
|  | Vegetable | ≥3 serving/day | 1289 (Cooked vegetables) 1299 (Salad or raw vegetables) |
|  | Whole grains | ≥3 serving/day | 1438, 1448 (Wholemeal or wholegrain bread) 1458, 1468 (Bran, oat, muesli cereal) |
|  | Fish | ≥2 serving/week | 1329 (Oily fish) 1339 (Non-oily fish) |
|  | Dairy | ≥2.5 serving/week | 1408 (Cheese) 1418 (Milk) |
|  | Refined grains | ≤1.5 serving/week | 1438, 1448 (Wholemeal or wholegrain bread) 1458, 1468 (Bran, oat, muesli cereal) |
|  | Processed meats | ≤1 serving/week | 1349 (Processed meat) 3680 (Age when last ate any kind of meat, 0 if indicated having never eaten meat) |
|  | Unprocessed meats | ≤1.5 serving/week | 1359 (Poultry) 1369 (Beef) 1379 (Lamb) 1389 (Pork) 3680 (Age when last ate any kind of meat, 0 if indicated having never eaten meat) |
|  | Sugar-sweetened beverages | ≤1 serving/week | 6144 (Never eats sugar or foods/drinks containing sugar) |
| Lifestyle behavior | Healthy | Having at least three healthy lifestyle factors | |
|  | Intermediate | Having two healthy lifestyle factors | |
|  | Unhealthy | Having one or fewer healthy lifestyle factor | |

**Supplemental Table 3. Hazard ratios and 95% confidential intervals for the cardiovascular risk according to age and sex category**

|  |  |  | **Crude** | | **Model 1** | | ***P* for interaction** | **Model 2** | | ***P* for interaction** |
| --- | --- | --- | --- | --- | --- | --- | --- | --- | --- | --- |
|  |  |  | **HR (95% CI)** | ***P* value** | **HR (95% CI)** | ***P* value** |  | **HR (95% CI)** | ***P* value** |  |
| Age | 40-55 years | Glaucoma (-) | Ref. |  | Ref. |  | 0.015 | Ref. |  | 0.117 |
|  |  | Glaucoma (+) | 1.79 (1.39-2.30) | <0.0001 | 1.44 (1.12-1.86) | 0.0041 |  | 1.33 (1.02-1.74) | <0.0001 |  |
|  | 56-70 years | Glaucoma (-) | Ref. |  | Ref. |  |  | Ref. |  |  |
|  |  | Glaucoma (+) | 1.29 (1.18-1.40) | <0.0001 | 1.12 (1.03-1.22) | 0.0091 |  | 1.11 (1.01-1.22) | 0.0254 |  |
| Sex | Men | Glaucoma (-) | Ref. |  | Ref. |  | <0.001 | Ref. |  | 0.001 |
|  |  | Glaucoma (+) | 1.38 (1.24-1.53) | <0.0001 | 1.04 (0.94-1.16) | 0.4071 |  | 1.04 (0.93-1.16) | 0.5398 |  |
|  | Women | Glaucoma (-) | Ref. |  | Ref. |  |  | Ref. |  |  |
|  |  | Glaucoma (+) | 1.89 (1.66-2.15) | <0.0001 | 1.35 (1.18-1.53) | <0.0001 |  | 1.32 (1.14-1.52) | 0.0001 |  |

Model 1: Age + Sex + Race

Model 2: Model 1 + Familial history of heart disease + Familial history of stroke + BMI + Current smoking + Physical activity + Eating habits

**Supplemental Table 4. Hazard ratios and 95% confidential intervals for the cardiovascular risk according to lifestyle behaviors**

|  | **Model 1** | ***P* value** |
| --- | --- | --- |
|  | **HR (95% CI)** |  |
| Glaucoma (-)/ Unfavorable lifestyle | Reference |  |
| Glaucoma (-)/ Intermediate lifestyle | 0.70 (0.67-0.73) | <0.001 |
| Glaucoma (-)/ Favorable lifestyle | 0.50 (0.48-0.52) | <0.001 |
| Glaucoma (+)/ Unfavorable lifestyle | Reference |  |
| Glaucoma (+)/ Intermediate lifestyle | 0.51 (0.40-0.64) | <0.001 |
| Glaucoma (+)/ Favorable lifestyle | 0.47 (0.38-0.58) | <0.001 |
| Glaucoma (-)/ Smoking (+) | Reference |  |
| Glaucoma (-)/ Smoking (-) | 0.57 (0.55-0.59) | <0.001 |
| Glaucoma (+)/ Smoking (+) | Reference |  |
| Glaucoma (+)/ Smoking (-) | 0.77 (0.60-0.99) | 0.042 |
| Glaucoma (-)/ Obese (+) | Reference |  |
| Glaucoma (-)/ Obese (-) | 0.64 (0.62-0.65) | <0.001 |
| Glaucoma (+)/ Obese (+) | Reference |  |
| Glaucoma (+)/ Obese (-) | 0.62 (0.52-0.73) | <0.001 |
| Glaucoma (-)/ Regular physical activity (-) | Reference |  |
| Glaucoma (-)/ Regular physical activity (+) | 0.79 (0.76-0.81) | <0.001 |
| Glaucoma (+)/ Regular physical activity (-) | Reference |  |
| Glaucoma (+)/ Regular physical activity (+) | 0.77 (0.65-0.91) | 0.002 |
| Glaucoma (-)/ Poor eating habits | Reference |  |
| Glaucoma (-)/ Good eating habits | 0.92 (0.88-0.96) | <0.001 |
| Glaucoma (+)/ Poor eating habits | Reference |  |
| Glaucoma (+)/ Good eating habits | 0.82 (0.63-1.06) | 0.128 |

Model 1: Age + Sex + Race

**Supplemental Table 5. Absolute risk for cardiovascular disease according to decile level of IOP in 103,156 participants whose IOP levels were available**

|  | Total | 1st | 2nd | 3rd | 4th | 5th | 6th | 7th | 8th | 9th | 10th | *P* for trends |  |
| --- | --- | --- | --- | --- | --- | --- | --- | --- | --- | --- | --- | --- | --- |
|  | (n=103156) | (n=10248) | (n=10404) | (n=10276) | (n=10278) | (n=10334) | (n=10350) | (n=10345) | (n=10316) | (n=10301) | (n=10264) |  |  |
| IOP, range (mmHg) |  | 0-11.4 | 11.4-12.8 | 12.8-13.8 | 13.8-14.8 | 14.8-15.7 | 15.7-16.6 | 16.6-17.6 | 17.6-18.9 | 18.9-20.8 | 20.8-126.2 |  |  |
| CVD events | 4323 (4.19) | 397 (3.87) | 380 (3.68) | 439 (4.22) | 378 (3.68) | 416 (4.03) | 425 (4.11) | 441 (4.26) | 479 (4.64) | 483 (4.69) | 485 (4.73) | <0.001 |  |

Data are n (%)

IOP, intraocular pressure; CVD, cardiovascular disease

**Supplemental Table 6. Absolute risk for cardiovascular disease according to decile level of intraocular pressure in 101,183 participants without baseline glaucoma whose IOP levels were available**

|  | Total | 1st | 2nd | 3rd | 4th | 5th | 6th | 7th | 8th | 9th | 10th | *P* for trends |
| --- | --- | --- | --- | --- | --- | --- | --- | --- | --- | --- | --- | --- |
|  | (n=101183) | (n=10152) | (n=10123) | (n=10096) | (n=10136) | (n=10133) | (n=10072) | (n=10129) | (n=10113) | (n=10122) | (n=10107) |  |
| IOP, range (mmHg) |  | 0.8-11.9 | 11.9-13.1 | 13.1-14.1 | 14.1-14.9 | 14.9-15.7 | 15.7-16.6 | 16.6-17.5 | 17.5-18.6 | 18.6-20.2 | 20.2-115.9 |  |
| CVD events | 4199 (4.15) | 389 (3.83) | 372 (3.67) | 421 (4.17) | 375 (3.70) | 406 (4.01) | 411 (4.08) | 431 (4.26) | 462 (4.57) | 470 (4.64) | 462 (4.57) | <0.001 |

Data are n (%)

IOP, intraocular pressure; CVD, cardiovascular disease

**Supplemental Table 7. Absolute risk for cardiovascular disease according to decile level of intraocular pressure in 1,973 participants with baseline glaucoma whose IOP levels were available**

|  | Total | 1st | 2nd | 3rd | 4th | 5th | 6th | 7th | 8th | 9th | 10th | *P* for trends |
| --- | --- | --- | --- | --- | --- | --- | --- | --- | --- | --- | --- | --- |
|  | (n=1973) | (n=198) | (n=197) | (n=197) | (n=198) | (n=197) | (n=197) | (n=198) | (n=196) | (n=197) | (n=198) |  |
| IOP, range (mmHg) |  | 6.0-13.6 | 13.7-15.5 | 15.5-16.6 | 16.6-17.7 | 17.7-18.6 | 18.6-19.8 | 19.8-21.0 | 21.0-22.4 | 22.4-24.8 | 24.8-57.9 |  |
| CVD events | 124 (6.28) | 17 (8.59) | 9 (4.57) | 12 (6.09) | 13 (6.57) | 13 (6.60) | 12 (6.09) | 18 (9.09) | 9 (4.59) | 11 (5.58) | 10 (5.05) | 0.624 |

Data are n (%)

IOP, intraocular pressure; CVD, cardiovascular disease

**
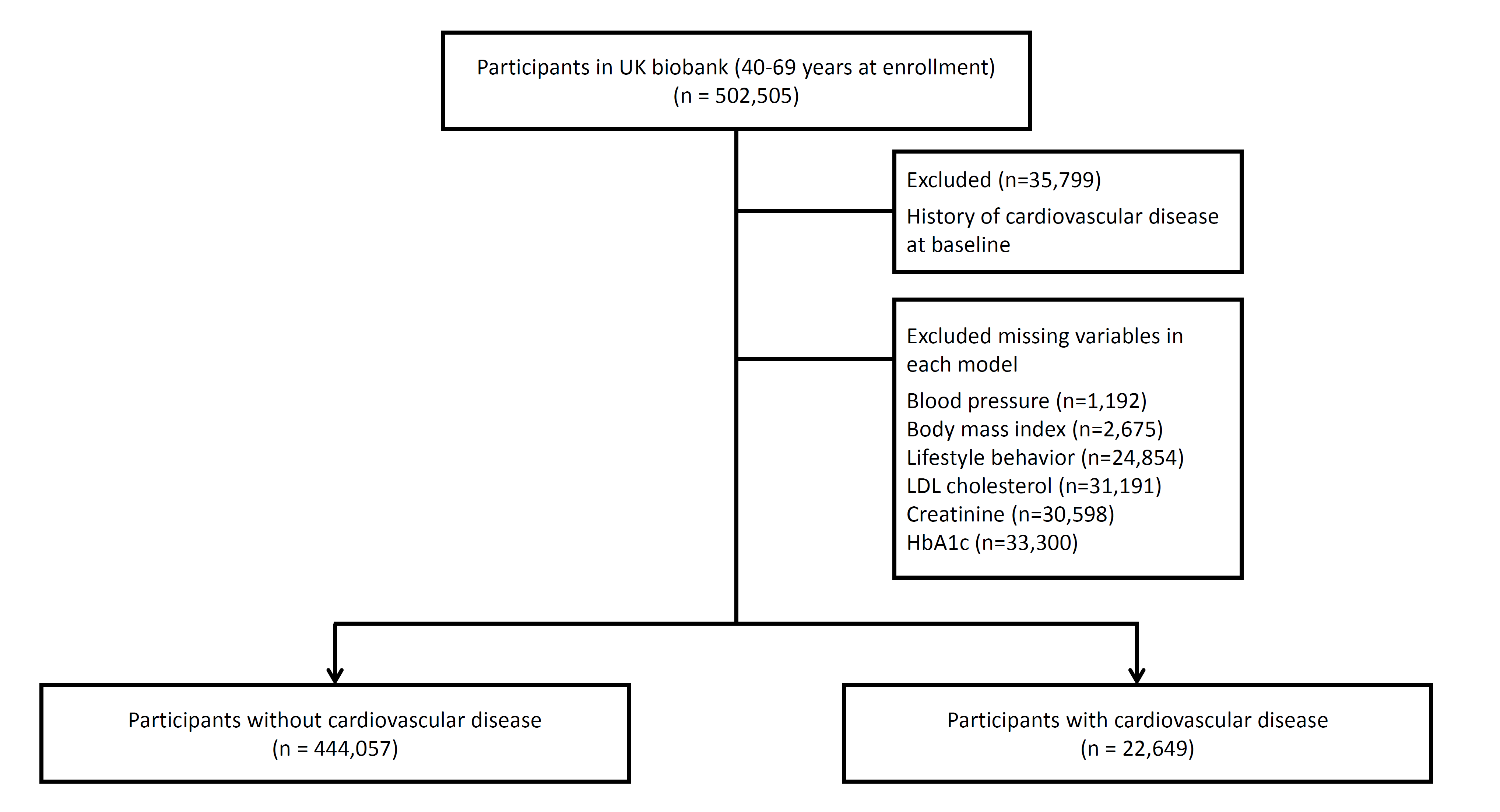
**

**Supplemental Figure 1. Study design summarizing enrollment and exclusion**
